# Home institution bias in the *New England Journal of Medicine*? A noninferiority study on citation rates

**DOI:** 10.1007/s11192-017-2584-7

**Published:** 2017-11-18

**Authors:** Alberto Falk Delgado, Anna Falk Delgado

**Affiliations:** 1 Department of Surgical Sciences, Plastic and Reconstructive Surgery, Uppsala University, Ing. 78/79 Plastikmottagningen, Akademiska Sjukhuset, 75185 Uppsala, Sweden; 2 Department of Clinical Neuroscience, Karolinska Institute, Stockholm, Sweden

**Keywords:** Citations rates, Institutional bias, Academic publishing, Impact of articles

## Abstract

Recently, in the four top journals of humanities, an institutional bias towards publication of authors from Harvard and Yale was shown. The *New England Journal of Medicine* (NEJM) is today the highest ranked general medical journal. It is unknown if there exists institutional bias favoring publication of articles originating from Harvard University, since the *NEJM* is produced by the Massachusetts Medical Society with close connections to the Harvard University. We examined if studies originating from the Harvard University published in the *NEJM* were noninferior in terms of citation rates compared to articles with an origin outside Harvard University. We evaluated original research articles published in the *NEJM* in 2000 up until June 2001. A two-sample noninferiority test based on the primary endpoint of citations was performed. Twenty-two studies were affiliated to the Harvard University and 280 studies were not affiliated to the Harvard University. The mean number of citations for Harvard affiliated studies was 625 (95% CI 358–952, median 354) and for non–Harvard affiliated studies 493 (95% CI 421–569, median 303). The mean difference was not statistically different between affiliations, but fulfilled the requirements for noninferiority [132 (95% CI − 138–402, *P* = 0.343), *Δ* 200]. In summary, citation rates were comparable between studies origination from the Harvard University compared to non-Harvard Institutions. Based on these results there appears to be low risk of institutional bias in the publishing process of original studies in the *NEJM.*

## Introduction

Scientific journals are ranked according to their impact factor (IF), where the IF is based upon the number of citations to that journals recent published articles divided by the number of published articles the two preceding years. According to this, the *New England Journal of Medicine* (NEJM) is today the highest ranked general medical journal, with a steadily increasing impact factor (IF) over the last 20 years that has now reached 72 in IF (Garfield 1996; Reuters 2017). This increasing IF might reflect that only the scientifically most rigorous and influential medical papers are published in the *NEJM.* Furthermore, 58% of the randomized controlled trials published in NEJM are entirely funded by a for-profit organization (Delgado and Delgado 2017).

The academic publication process in the *NEJM* should optimally ensure publication of only the most impactful papers, and the base for publication decision should rely primarily on scientific merit and novelty. Recently, in the four top journals of humanities, an institutional bias towards publication of authors from Harvard and Yale was shown (Piper and Wellman 2017). A finding of possible institutional inequality in the process of academic publishing. The *NEJM implemented a* policy *Too close to call* in 2001 to prevent “insider bias” among the editors (Curfman and Drazen 2001), when editors submitted work to their own journal. However, it is unclear if there exists institutional bias favoring publication of articles originating from Harvard University, since the *NEJM* is produced by the Massachusetts Medical Society with close connections to the Harvard University. How can one determine that only the most influential medical articles are published in the *NEJM*? One method to assess the impact of a paper is simply by determining its citation rate, rather than quality assessment methods (Wang et al. 2014). In this study, we examined if studies originating from the Harvard University published in the *NEJM* were noninferior in terms of citation rates compared to articles with an origin outside Harvard University.

## Methods

We evaluated original research articles published in the *NEJM* during 2000 and 2001. Articles from year 2000 and 2001 were chosen in order to have a fair chance of being cited up until 2017. Brief reports were excluded, since brief reports in general are less cited compared to original articles (unpublished results). The articles were gathered consecutively and manually from the webpage www.nejm.org. Institutional affiliation based on first and last author were registered and categorized as Harvard or non-Harvard affiliation. The affiliation to Harvard of either the first or last author categorized the research as pertaining to Harvard. Citation rates, as of August 2017, for each article were retrieved from www.nejm.org. Data was extracted by one author. Study sample size was determined through power calculation based on pilot data from the first 3 months of 2000 in the *Journal*. A two-sample noninferiority test based on the primary endpoint of citation rates was performed using a power of 90% with a 2.5% error rate and a delta value of 200. Implementing a sampling ratio of 5 the numbers of articles needed in each group was 14 articles from the Harvard University and 280 non-Harvard based studies. We estimated that we needed to include articles from January 2000 up to June 30 2001. A two-sample noninferiority test based on the primary endpoint of citations was performed with a power of 90% and type I error rate of 2.5% with non-inferiority (*Δ*) set to 200 citations. Non-parametric Mann–Whitney’s U test was used to compare differences between continuous variables. Analyses were performed in STATA Version 13.1 (StataCorp, USA). Descriptive summary statistics were presented with 95% confidence interval.

## Results

**Fig. 1:**
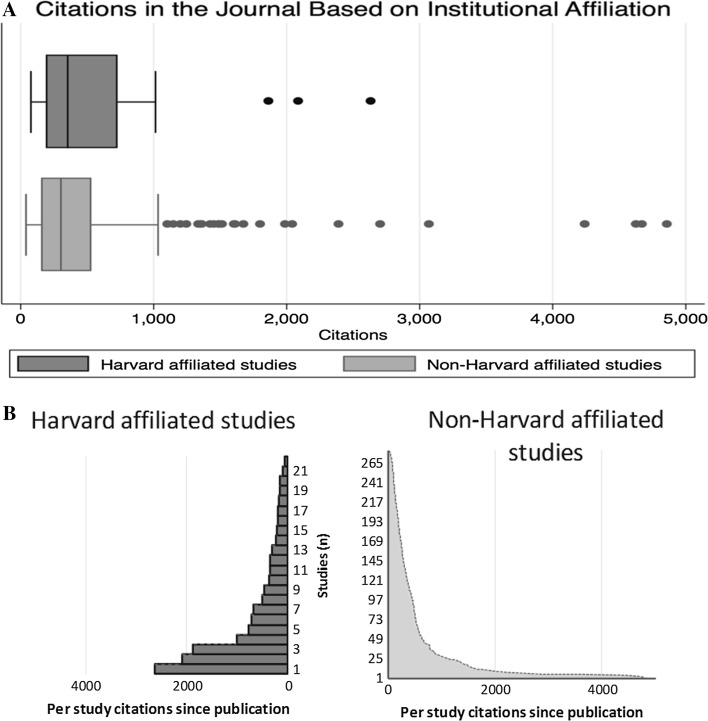
**a** Box and whiskers plot show median citations indicated by the vertical line and the interquartile range indicated by the box, with whiskers containing 1.5 times the interquartile range and outliers indicated by individual dots. Citation data is stratified for institutional affiliation: Harvard or non-Harvard. **b** Back to back histogram show the distribution of citations stratified for institutional affiliation: Harvard or non-Harvard. Each histogram depicts the actual per study citations with 22 Harvard affiliated studies on the left side and 280 non-Harvard affiliated studies on the right side distributed on the *y* axis

A total of 302 original articles published in the *NEJM* year 2000 up until June 2001 was identified. Twenty-two studies were affiliated to the Harvard University and 280 studies were not affiliated to the Harvard University. The mean number of citations for Harvard affiliated studies was 625 (95% CI 358–952, median 354, Fig. 1a, b) and for non–Harvard affiliated studies 493 (95% CI 421–569, median 303). The mean difference was not statistically different between affiliations, but fulfilled the requirements for noninferiority (132 (95% CI − 138–402, *P* = 0.343), *Δ* 200).

## Discussion

In total, eight percent of all articles published in the *NEJM* had a first or last author affiliated to the Harvard University. Based on these results there appears to be a low risk of institutional inequality in terms of impact of the articles published in *NEJM* publications from the Harvard University compared to publications to other institutions.

### Discuss the findings

Data from the humanities have suggested an institutional bias with a higher proportion of the authors affiliated to Yale and Harvard more likely to publish in the top ranked journals (Piper and Wellman 2017). Previous data from the humanities does however not reveal if the studies originating from Yale or Harvard are inferior compared to other institutions. Our study reveal that eight percent of *NEJM* publications are affiliated to Harvard, this skewed proportion with origin from *NEJMs* home institution might suggest evidence for institutional equality. Previous studies related to citation rates have focused on factors related to higher citation, where e.g. for-profit funded research and articles related to cancer/cardiovascular disease are associated with higher citation rates (Kulkarni et al. 2007), and length of the title (Jacques and Sebire 2010). Currently, few reports have assessed the influence of home institution bias in the editorial process (Weller 2002). Previous research has provided support for a national institutional publication bias, were authors or referees from the same country are favored for publication (Daniel 1993). Studies from Harvard were non-inferior and not superior to studies not affiliated to Harvard in terms of citations. This suggest that only the most impactful articles actually are published in *NEJM* despite its home institution. Furthermore, this study ensure a rigid editorial process for the acceptance of articles in *NEJM*.

### Strengths and limitations

The strength of this study is pertinent to the fact that the study was powered for the primary endpoint—noninferiority in citation rates. This is to our knowledge the first study to assess home institutional bias related to citation rates. Furthermore, we chose articles published from 2000 and 2001 to ensure a sufficient time to be cited, and less vulnerable to trends in citing. Few general medical journals are so strongly linked as *NEJM* to a particular institution. It is unclear if these finding can be generalized to other fields, or more specialized journals.

## Conclusion

We conclude that eight percent of the studies in *NEJM* were associated with Harvard University. However, we found no evidence of home institution bias between studies affiliated to Harvard and those not affiliated to the Harvard University with regards to citations rates.
